# Notes and News

**Published:** 1905-05-27

**Authors:** 


### Notes and News.

The reported outbreak of cerebro-meningitis near Northampton is apparently at an end.

The Westminster Hospital Bazaar, which closed on Thursday, was a brilliant success, and it is to be hoped has reaped a good harvest for the institution.

On the occasion of the King's visit to Aldershot on Wednesday he unveiled the memorial to the officers and men of the Royal Army Corps who fell in South Africa. The memorial is in the form of a granite obelisk.

A garden fête and sale of work, in aid of the London Homœopathic Hospital, will be held at the residence of Mr. R. Perks, M.P., 11 Kensington Palace Gardens, on Thursday, June 8. The fête is being arranged by the Ladies' Guild of the hospital. The Countess Cawdor will receive purses of £5 and upwards from little children.

The Japanese form of wrestling has evidently come to stay. A school has been opened in Oxford Street where four clever Japanese instructors are now busily occupied in trying to teach their art to pupils they must find singularly inept in comparison to those of their own country. Ju-jitsu, as it is called, and pronounced jew-jitss, is the triumph of skill and agility over strength, and is the outcome of a scientific knowledge of human anatomy and movement. Thus one skilled in ju-jitsu has a far more powerful but uninitiated opponent entirely at his mercy. The practice of ju-jitsu is not of necessity very fatiguing. It is only rendered so by the mistaken application of strength, which is the pitfall of the novice. A good many society ladies are taking up ju-jitsu, and so it will probably shortly become a fashionable pastime. Medical men also lend their approval to the exercise, and at a recent exhibition at the school several well-known members of the medical profession were interested spectators.

The choice of a site for the proposed consumption sanatorium for Cork is still causing difficulty.

Lord Strathcona and Mount Royal will preside at the Festival Dinner in aid of the National Hospital for the Paralysed and Epileptic at the Hôtel Métropole on Thursday, June 15th.

A new medical paper called *The Anti-Septic* is now published in Madras. The editor is Dr. T. Nair, M.D.Edin., and the proprietor is U. Rama Rai, who describes himself as a medical practitioner.

A fever hospital has been opened at Linlithgow for Linlithgow district and burgh. It contains 25 beds in two pavilions, with administration block and the usual offices. The cost is estimated at about £9,000.

Messrs. Howell and James have generously provided offices, free of cost, for the use of the Special Appeal Committee of the Royal Waterloo Hospital for Children and Women, which is endeavouring to raise £20,000 for building expenses, and to increase the permanent income.

A grand Café Chantant will be held on May 30th in the Empire Room, Trocadero Restaurant, in aid of the Great Ormond Street Hospital for Children. A large number of prominent artistes have given their services. Tickets, 6s. each, can be obtained from Mr. F. Brighten, the director, 180 Ladbroke Grove.

The desirability of concentrating at a few centres the teaching of the preliminary and intermediate subjects of the medical curriculum in London is a matter which has long occupied the attention of those interested in medical education. It has been felt that such a step would certainly bring greater efficiency in teaching, as well as economy in expenditure. The Westminster Hospital Medical School has been the first to take definite action in the matter, and has just completed negotiations with King's College by which arrangements have been made for the teaching of physics, chemistry, anatomy, physiology, and materia medica (that is to say, the subjects of the Preliminary and Intermediate Examinations) to Westminster students at King's College. Students will enter Westminster Hospital Medical School as in the past, and will remain Westminster men and will not become matriculated students of King's College; but they will be taught the earlier subjects of medical study at that institution. The scheme will come into effect at the commencement of next Winter Session in October. At the same time the Westminster School is thoroughly reorganising the teaching of the subjects of the final examination. This arrangement, though practically completed before it was issued, is in accord with the views expressed in the recent report of the King Edward Hospital Fund Committee, and it is believed that this commencement of a probably more general concentration of the teaching of the preliminary and intermediate subjects of the curriculum cannot but promote the best interests of medical education in London.

